# Ultrasound assessment of the respiratory system using diaphragm motion-volume indices

**DOI:** 10.3389/fmed.2023.1190891

**Published:** 2023-05-19

**Authors:** Alain Boussuges, Guillaume Chaumet, Martin Boussuges, Amelie Menard, Stephane Delliaux, Fabienne Brégeon

**Affiliations:** ^1^Center for Cardiovascular and Nutrition Research, C2VN (Aix Marseille University, INSERM 1263, INRAE 1260), Faculté de Médecine, Marseille, France; ^2^APHM, Hôpital Nord, Explorations Fonctionnelles Respiratoires, Marseille, France; ^3^AltraBio, Lyon, France; ^4^Aix Marseille University, APHM, Département de Pneumologie, Hôpital Nord, Marseille, France; ^5^APHM, Hôpital Nord, Unité Post COVID, Service de Médecine Interne, Marseille, France; ^6^Aix Marseille University, APHM, Microbes Evolution Phylogeny and Infections Department, IHU-Méditerranée Infection, Marseille, France

**Keywords:** chest ultrasonography, hemidiaphragm, thickening fraction, thickening ratio, diaphragm function

## Abstract

**Background:**

Although previous studies have determined limit values of normality for diaphragm excursion and thickening, it would be beneficial to determine the normal diaphragm motion-to-inspired volume ratio that integrates the activity of the diaphragm and the quality of the respiratory system.

**Methods:**

To determine the normal values of selected ultrasound diaphragm motion-volume indices, subjects with normal pulmonary function testing were recruited. Ultrasound examination recorded diaphragm excursion on both sides during quiet breathing and deep inspiration. Diaphragm thickness was also measured. The inspired volumes of the corresponding cycles were systematically recorded using a spirometer. The indices were calculated using the ratio excursion, or percentage of thickening, divided by the corresponding breathing volume. From this corhort, normal values and limit values for normality were determined. These measurements were compared to those performed on the healthy side in patients with hemidiaphragm paralysis because an increase in hemidiaphragm activity has been previously demonstated in such circumstances.

**Results:**

A total of 122 subjects (51 women, 71 men) with normal pulmonary function were included in the study. Statistical analysis revealed that the ratio of excursion, or percentage of thickening, to inspired volume ratio significantly differed between males and females. When the above-mentioned indices using excursion were normalized by body weight, no gender differences were found. The indices differed between normal respiratory function subjects and patients with hemidiaphragm paralysis (27 women, 41 men). On the paralyzed side, the average ratio of the excursion divided by the inspired volume was zero. On the healthy side, the indices using the excursion and the percentage of thickening during quiet breathing or deep inspiration were significantly increased comparedto patients with normal lung function. According to the logistic regression analysis, the most relevant indice appeared to be the ratio of the excursion measured during quiet breathing to the inspired volume.

**Conclusion:**

The normal values of the diaphragm motion-volume indices could be useful to estimate the performance of the respiratory system. Proposed indices appear suitable in a context of hyperactivity.

## Introduction

It is now recognized that ultrasonography is an accurate tool for assessing diaphragm function (1). Diaphragm motion can be recorded using M-mode ultrasonography during various maneuvers such as quiet breathing, voluntary sniffing and deep breathing i.e. deep inspiration to total lung capacity (TLC) (2–6). In addition, it is possible to measure the diaphragm thickness at the area of apposition of the diaphram to the rib cage, at the end of expiration and inspiration (5, 7, 8). These measurements allow calculating the percentage of inspiratory thickening both during quiet breathing and deep inspiration.

Thanks to these various procedures, it is possible to detect dysfunction of the diaphragm. Hemidiaphragm paralysis is diagnosed using some characteristics such as lack of movement or paradoxical movement during quiet breathing (9, 10). In addition, a paradoxical motion is recorded during sniffing and at the early phase of a deep inspiration. To support the diagnosis of paralysis, measuring diaphragm thickness and thickening is useful. In patients with hemidiaphragm paralysis, thining or low thickening (thickening fraction less than 20%) is recorded during deep inspiration (11). The lower limit of normality for diaphragm thickness measured at the end of expiration (12) can be used to detect diaphragm atrophy in patients with long-term hemidiaphragm paralysis.

Diagnosing dysfunction without complete paralysis is more challenging. Some criteria can be used such as an excursion during deep inspiration below normal values. Since excursions vary with body position (13), normal values should be used according to the examination conditions, i.e., supine, sitting, or standing. Furthermore, the diagnosis of diaphragm dysfunction is supported by the study of thickening fraction. In patients suffering from moderate or severe diaphragm dysfunction, thickening fraction is lower than 60 and 40%, respectively (14).

The above mentioned criteria are particularly interesting for detecting diaphragm dysfunction and paralysis. On the other hand, additional parameters able to estimate diaphragm efficiency would be usefull, especially when confounding factors affecting diaphragm motion coexist as in case of respiratory diseases with altered lung compliance, neuromuscular disorders or during weaning from mechanical ventilation. Various lung diseases have been shown to alter the anatomy and function of the diaphragm. In COPD patients, diaphragm biopsies have reported changes in fiber type, weakness in diaphragm muscle fibers and loss of myosin and contractile proteins (15). Diaphragm ultrasonography has reported, during rest breathing, increased diaphragm excursion in COPD patients compared to healthy controls (16). Conversely, excursions are decreased during deep inspiration (16). A relationship between the severity of COPD, assessed by the pulmonary function testing (PFT) or the decrease in exercise capacity, and the limitation of the excursion during deep inspiration has been reported (17–21).

Furthermore, a decrease in diaphragm thickness, measured at the zone of apposition of the diaphragm to the rib cage, has been reported in mild and moderate COPD patients (22). In contrast, an increase in diaphragm thickness was reported in severe COPD (22). To explain this finding, it was suggested that hypoxia-induced hyperventilation, in the most severe cases, lead to an increase in diaphragm activity.

Similar observations were made in patients with interstitial lung disease. Indeed, it has been reported, a thicker diaphragm in such patients compared to healthy controls, supporting the hypothesis of a thickening of the diaphragm in response to respiratory muscle overload (23).

Early detection of diaphragm hyperactivity should be helpful to assess the severity of lung parenchymal diseases and better explaining clinical disorders such as dyspnea and exercise capacity limitation.

In this context, to estimate the performance of the respiratory system integrating diaphragmatic function and the quality of the thoraco-pulmonary system, it would be useful to determine the normal relationship between diaphragm motion and inspired volume. Therefore, we first studied a group of subjects with normal lung function to determine the normal ratios between diaphragm excursion, or thickening, and inspired volumes.

An hyperactivity of the hemidiaphragm should increase these indices. To support this hypothesis, measurements performed in subjects with normal lung function were compared with those recorded on the healthy side in patients with unilateral hemidiaphragm paralysis.

Indeed, it is recognized that, in such patients an increase in the neural tract develops on the healthy side to compensate the decrease in diaphragm efficiency secondary to the paralysis of an hemidiaphragm (10, 24–27).

## Materials and methods

This study fell within the French legal framework of non-interventional research as performed on medical data collected during standard clinical care, and was not considered as study involving human beings (Jardé law Article L1121-1 of December 31, 2016). The research was approved by the ethics board of the Marseilles Hospital institution (registered under number 2RLK6P).

The first objective of the study was to determine the normal value of various diaphragm motion-volume indices. Second, the results from subjects with normal lung function were compared to measurements in a sample of patients suffering from known hemidiaphragm paralysis.

### Subjects with normal lung function

The subjects with normal lung function were recruited from the medical consultation of the Pulmonary Function Testing unit of the North hospital in Marseilles carried out from June 2020 to October 2022. Subjects were considered to have normal lung function when they had no prior history of respiratory disorders, a normal chest imaging, no clinical disorders at the time of examination and a normal lung function test. Standard pulmonary function tests (PFTs) were performed using a spirometer (PFT MasterLab Jaeger plethysmograph, Bunnik, Netherlands) according to the ERS/ATS standards (28). The criteria for classifying the pulmonary function test as normal were a slow vital capacity (SVC), a forced vital capacity (FVC) and a forced expiratory volume in 1sec (FEV1) larger than the lower limit of normal for the reference population, and a FEV1 to FVC ratio above 0.7.

### Patients with hemidiaphragm paralysis

These patients were sent to the respiratory function laboratory with a previous diagnosis of hemidiaphragm paralysis. This diagnosis was the more frequently performed by another medical team using conventional methods including PFTs, fluoroscopy and diaphragm electromyography (29) and/or clinical context (surgery with phrenic nerve section for exemple). It is recognized that pleural effusion can impair diaphragm motion, sometimes leading to an aspect of hemidiaphragm paralysis. Consequently, patients with pleural effusion, on the paralyzed side or on the healthy side, were excluded.

At the time of our study, it was verified by our ultrasound examination that hemidiaphragm paralysis was still present using previously published criteria (10). Measurement of diaphragm motion on the healthy side was performed as described in subjects with normal lung function.

### Calculation of the sample

Calculating the number of patients to be included was performed for the comparison between patients with normal lung function and patients with hemidiaphragm paralysis. In a previous work (10), it was reported that in patients with hemidiaphragm paralysis, excursions were increased on the healthy side during quiet breathing compared to healthy controls (3 ± 1.1 vs. 1.8 ± 0.3 cm; *p* < 0.001). To find differences between the ratio excursion/inspired volume in patients and controls, the sample calculation was based on this study. For a 0.05% risk alpha and a 95% power, it was calculated that at less 22 subjects should be included in each group.

For the determination of normal values, the largest number of subjects with normal lung function was recruited. Considering that some parameters such as excursion or thickness during deep inspiration may be difficult to measure, a target sample size of around 100 subjects with normal lung function was targeted.

### Ultrasound study

The ultrasonographic examinations were carried out by an experienced investigator (more than 1000 diaphragm ultrasound examinations performed prior to the beginning of the study) using commercially available ultrasound machine (Vivid S60N, GE Medical System, Milwaukee, Wl, USA) connected to a 1.5–3 MHz transducer array (3Sc probe) for excursion measurements and to a linear vascular transducer (9L probe) for thickness measurements.

#### Assessment of diaphragm excursion

The patients were investigated while sitting and diaphragm excursions were assessed by M-mode ultrasonography using a previously published method (30). A subcostal or low intercostal probe position was chosen between the midclavicular and posterior axillary lines in order to obtain the best imaging of both hemidiaphragmatic domes in two-dimensional mode (B-mode). When the approach of the hemidiaphragm was considered good and the exploration line was perpendicular to the posterior part of each hemi-diaphragm, M-mode was used to record the diaphragm displacement. To an accurate perpendicular approach, anatomical M-mode was used. Diaphragmatic motion was assessed while breathing at tidal volume (quiet breathing). In addition, diaphragmatic excursions were measured during deep inspiration (from functional residual capacity to total lung capacity). All ultrasound studies have been saved for subsequent blind analysis. The diaphragmatic inspiratory excursions were measured by placing the first caliper at the foot of the inspiration slope on the diaphragmatic echo line and placing the second caliper at the top of the curve. For rest breathing, the velocities of displacement (excursion divided by inspiration time) were also measured. For deep inspiration, several maneuvers were recorded, and the maximal excursion, i.e., the greatest distance between the baseline and the apex, was retained. For each diaphragm excursion, the corresponding inspired volume was recorded.

#### Assessment of diaphragm thickening

Diaphragmatic thickness was assessed by B-mode ultrasonography using a previously published method (14). Both hemidiaphragms were visualized at the zone of apposition, and the probe was placed below the phrenico-costal sinus near the anterior or mid-axillary line at the eighth or ninth intercostal space. The diaphragm was identified as a three-layered structure with two parallel echogenic lines, the diaphragmatic pleura and the peritoneal fascia, enclosing the hypoechoic diaphragmatic muscle. A third hyperechoic line was frequently observed in the middle of the non-echogenic layer, considered to be the fibrous layer in the center of the diaphragm. The intercostal space that provided the best visualization of the diaphragm was chosen and the probe was positioned so that the two lines delimiting the diaphragm were parallel. The thickness of each hemidiaphragm was directly measured from the frozen B-mode images. The diaphragm thickness was measured as the distance between the pleural membrane and the peritoneal membrane, without including these lines in the measurement (31). The measurements were made at end-expiratory time (when the lung was filled at the functional residual capacity), at the end of inspiration during quiet breathing at tidal volume, and after deep inspiration up to total lung capacity (TLC). The percentage of thickening, i.e., the difference between end-inspiration and end-expiration thickness divided by the thickness at end-expiration, was determined for both hemidiaphragms during quiet breathing and during deep inspiration.

#### Measurements of ultrasound-synchronized inspiratory volumes

The measurements of the inspiratory volumes were carried out breath by breath using a device developped in our laboratory that can be connected to the ultrasound machine (32). Continuous monitoring of the gas volume was performed using a commercially available turbine spirometer (Flow Mir, Medical International Research, Rome, Italy). The turbine support incorporated 3 sensors (1 infrared led and 2 infrared phototransistors) to detect the change in the respiratory cycle (inspiration, expiration) and measure the gas volume. Inspiratory volume and breathing rate were displayed on an LCD screen. We focused on inspired volume during quiet breathing at tidal volume (Vti) and on the inspired volume during a deep inspiration (Vimax). In addition, electronic signals corresponding to the beginning of the breathing time, i.e., inspiration and expiration, were sent to the ultrasound machine via ECG cables.

#### Calculation of ultrasound diaphragm motion-volume indices

Various parameters combining inspired volumes and the corresponding diaphragm motion were choosen as they can inform on diaphragm efficacy and thoraco-pulmonary system. These indices were calculated from measurements performed in both volunteers with normal lung function and patients suffering from hemidiaphragm paralysis and were during quiet breathing:

- Diaphragm excursion (in mm) divided by Vti (in L) (Inspiratory excursion (QB)/Vti).
- Diaphragm inspiratory velocity (in mm/s) divided by Vti (in L) (Inspiratory velocity (QB)/Vti).
- Percentage of inspiratory thickening (in %) divided by Vti (in L) (Inspiratory thickening (QB)/Vti).

Calculations were also performed during deep inspiration with the diaphragm excursion (in mm) or the thickening fraction (in %) divided by Vimax (in L) (Inspiratory excursion (DI)/Vimax and Thickening fraction (DI)/Vimax).

The same calculations were made with the inspired volume divided by body mass (in ml kg^–1^) in order to obtained indexed parameters.

### Statistical analysis

The results are reported as means ± SD. The lower and upper limits of normal for the ultrasound markers of diaphragm activity were calculated as the 5th percentile and 95th percentile, repectively.

The measurements performed in the subjects with normal lung function were compared with the healthy side of the patients with hemidiaphragm paralysis. Comparisons were performed using unpaired Student’s *t*-test when the variables were normally distributed. When the variables were not normally distributed, comparisons were performed with the Mann–Whitney U-test. Thereafter, a logistic regression model was computed to find the more relevant ultrasound indice for the prediction of increased diaphragm activity. The categorical variable was based on the chracteristics of the two populations studied:

- Increase in diaphragm activity in patients with contralateral hemidiaphragm paralysis.
- No increase in diaphragm activity in patients with normal lung function.

Some indices varied according to sexe, consequently men and women were analyzed separately.

Statistical analyses were run on R statistical software. Differences between groups were considered significant at *p* < 0.05.

## Results

### Populations studied

#### Subjects with normal lung function

**FIGURE 1:**
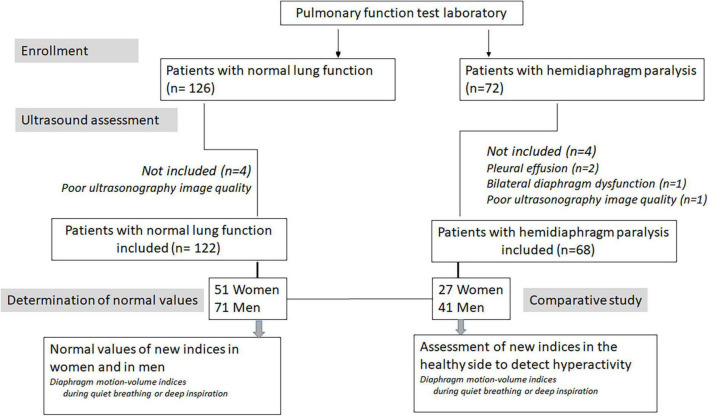
Flow diagram showing the enrollment of patients participating in the study.

To begin with, 126 subjects with normal pulmonary function were initially screened and 122 were ultimately included in the study (Figure 1). Their characteristics are reported in Table 1. In nine cases, the complete excursion of the left hemidiaphragm could not be measured. All other ultrasound parameters could be accurately assessed by the investigator.

**TABLE 1:** Anthropometric data and standard PFT results in subjects with normal lung function (NLF) and patients with hemidiaphragm paralysis (HP).

	Subjects with NLF	Patients with HP	*p*-Value
Women/men	51–71	27–41	NS
Age	50 ± 16	54 ± 14	NS
Height	171 ± 6	170 ± 9	NS
Weight	72 ± 14	72 ± 15	NS
SVC (L)	4.2 ± 1.2	2.8 ± 1.2	<0.001
SVC (% of predicted)	99 ± 13	70 ± 15	<0.001
FVC (L)	4.1 ± 1.2	2.7 ± 0.9	<0.001
FVC (% of predicted)	99 ± 14	71 ± 16	<0.001
FEV_1.0_ (L)	3.3 ± 0.9	2 ± 0.7	<0.001
FEV_1.0_ (% of predicted)	97 ± 16	67 ± 15	<0.001
FEV_1.0_/FVC	79 ± 11	76 ± 10	NS

#### Patients with hemidiaphragm paralysis

In the population of patients with hemidiaphragm paralysis, 72 patients were initially recruited from the medical consultation and 68 patients were definitively included (see Figure 1 and Table 1). The context of the discovery of the hemidiaphragm paralysis was a recent thoracic or cervical surgery in 38 cases, recent trauma in 6 cases, thoracic cancer in 4 cases, recent viral infection in 3 cases, neuromuscular disease in 3 cases, and unexplained dyspnea with elevated hemidiaphragm on chest X-ray in 14 cases. The paralysis was on the right side in 31 cases, on the left side in 37 cases. Decreased vital capacity was observed in patients with hemidiaphragm paralysis (Table 1).

### Ultrasound measurements

Table 2 reports the ultrasound measurements performed in patients with normal lung function (NLF). As expected, excursion and thicknesses were increased in men in comparison with women.

**TABLE 2:** Measurements of hemidiaphragm excursions and thicknesses in subjects with NLF.

	Women	Men	*p*-Value
	**Mean ± SD**		
**Right hemidiaphragm**			
Inspiratory excursion (QB) (cm)	1.85 ± 0.4	2.07 ± 0.5	0.01
Inspiratory velocity (QB) (cm/s)	1.75 ± 0.6	1.93 ± 0.76	NS
Inspiratory excursion (DI) (cm)	4.76 ± 0.9	5.85 ± 0.9	<0.001
Expiratory thickness (mm)	1.76 ± 0.44	2.1 ± 0.47	<0.001
Inspiratory thickening (QB) (%)	35 ± 18	34 ± 16	NS
Thickening fraction (DI) (%)	115 ± 42	110 ± 43	NS
**Left hemidiaphragm**			
Inspiratory excursion (QB) (cm)	1.95 ± 0.47	2.07 ± 0.5	NS
Inspiratory velocity (QB) (cm/s)	1.94 ± 0.72	1.91 ± 0.57	NS
Inspiratory excursion (DI) (cm)	4.93 ± 0.8	6.09 ± 0.87	<0.001
Expiratory thickness (mm)	1.61 ± 0.31	1.94 ± 0.39	<0.001
Inspiratory thickening (QB) (%)	33 ± 15	30 ± 16	NS
Thickening fraction (DI) (%)	112 ± 30	111 ± 38	NS
Right to left ratio of excursion (DI)	0.98 ± 0.17	0.98 ± 15	NS

NLF, normal lung function; QB, quiet breathing; DI, deep inspiration.

Table 3 reports the result of the diaphragm motion-volume indices. Vti was higher in men than in women: respectively 0.67L ± 0.17 vs. 0.49L ± 0.17 during the study of the right hemidiaphragm and 0.65 ± 0.15 L vs. 0.49 ± 0.17L for the left hemidiaphragm. The differences between men and women were highly significant for both sides (*p* < 0.001). These differences (*p* < 0.001) were also recorded for Vimax (3 ± 0.7 L vs. 2 ± 0.6L for right side and 2.9 ± 0.8 vs. 2 ± 0.6L for left side in men and women, respectively.

**TABLE 3:** Diaphragm motion-volume indices in subjects with normal lung function.

	Women	Men	*p*-Value
	**Mean ± SD [5th–95th percentiles]**		
**Right hemidiaphragm**			
Inspiratory excursion (QB)/Vti (mm/L)	41 ± 12 [25–62]	32 ± 8 [22–46]	<0.001
Inspiratory velocity (QB)/Vti (mm/s/L)	39 ± 17 [17–72]	30 ± 11 [16–52]	<0.001
Inspiratory excursion (DI)/Vimax (mm/L)	26 ± 7 [17–38]	21 ± 5 [13–28]	<0.001
Inspiratory thickening (QB)/Vti (%/L)	75 ± 38 [22–136]	53 ± 25 [17–92]	<0.001
Thickening fraction (DI)/Vimax (%/L)	62 ± 24 [28–109]	39 ± 17 [17–69]	<0.001
**Left hemidiaphragm**			
Inspiratory excursion (QB)/Vti (mm/L)	43 ± 15 [22–65]	33 ± 9 [23–49]	<0.001
Inspiratory velocity (QB)/Vti (mm/s/L)	42 ± 19 [21–80]	31 ± 11 [17–48]	<0.001
Inspiratory excursion (DI)/Vimax (mm/L)	27 ± 7 [16–40]	22 ± 5 [14–31]	<0.001
Inspiratory thickening (QB)/Vti (%/L)	70 ± 35 [34–136]	49 ± 29 [21–116]	<0.001
Thickening fraction (DI)/Vimax (%/L)	61 ± 21 [32–94]	40 ± 17 [18–73]	<0.001
Right to left ratio Inspiratory excursion (DI)/Vimax	0.99 ± 0.18 [0.7–1.3]	0.96 ± 15 [0.7–1.2]	NS

Vti, inspired volume during quiet breathing at tidal volume; Vimax, inspired volume during a deep inspiration; QB, quiet breathing; DI, deep inspiration.

**FIGURE 2:**
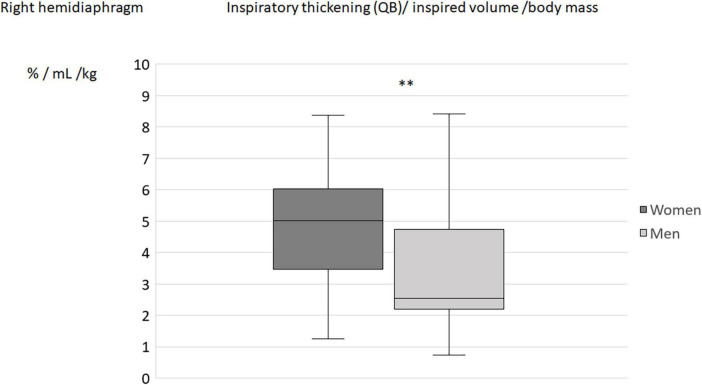
Inspiratory thickening during quiet breathing (QB) divided by inspired volume related to the body mass in men and women: Results on the right hemidiaphragm. **p < 0.05.

**FIGURE 3:**
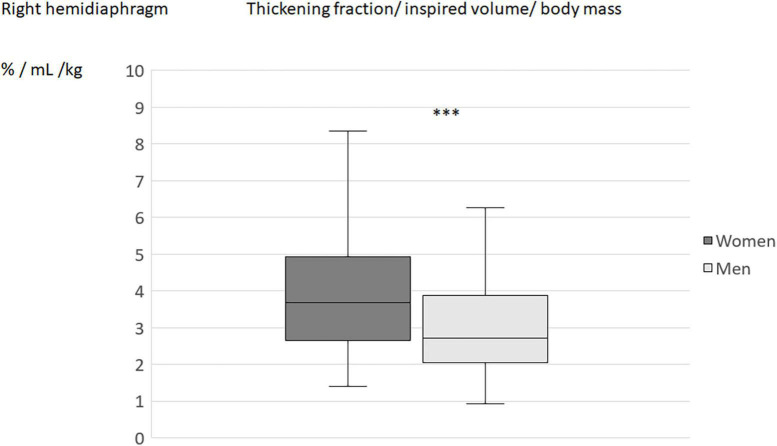
Thickening fraction divided by inspired volume related to the body mass in men and women: Results on the right hemidiaphragm. ***p < 0.001.

**FIGURE 4:**
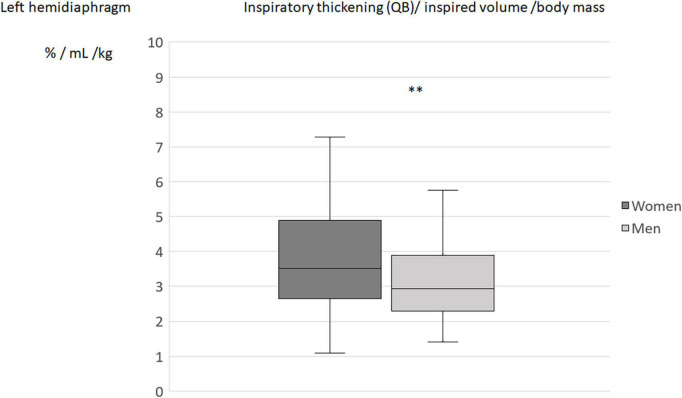
Inspiratory thickening during quiet breathing (QB) divided by inspired volume related to the body mass in men and women: Results on the left hemidiaphragm. **p < 0.05.

**FIGURE 5:**
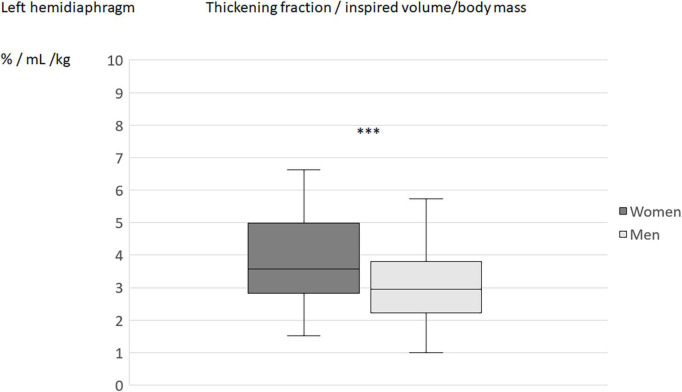
Thickening fraction divided by inspired volume related to the body mass in men and women: Results on the left hemidiaphragm. ***p < 0.001.

When the diaphragm excursion, during quiet breathing or deep inspiration, was divided by the inspired volume indexed by the subject’s body weight, no differences in the indices was found between men and women (Table 4). On the other hand, the differences between sexes remained significant for the indices using the percentage of thickening (during quiet breathing or deep inspiration) for both hemidiaphragms (Figures 2–5).

**TABLE 4:** Diaphragm motion-volume indices in subjects with normal lung function (in Vol/kg).

	Women	Men	*p*-Value
**Mean ± SD [5th–95th percentiles]**			
**Right hemidiaphragm**			
Inspiratory excursion (QB)/Vti (mm/ml/kg)	2.6 ± 0.8 [1.5–3.8]	2.5 ± 0.8 [1.5–4]	NS
Inspiratory velocity (QB)/Vti (mm/s/L/kg)	2.59 ± 1.3 [1.1–4.9]	2.38 ± 1 [1.2–4]	NS
Inspiratory excursion (DI)/Vimax (mm/ml/kg)	1.66 ± 0.5 [1–2.7]	1.6 ± 0.5 [1–2.2]	NS
Inspiratory thickening (QB)/Vti (%/L/kg)	4.8 ± 2.4 [1.5–8.3]	4 ± 1.8 [1.4–6.6]	0.03
Thickening fraction (DI)/Vimax (%/L/kg)	3.9 ± 1.6 [1.9–6.6]	3 ± 1.4 [1.4–5.5]	<0.001
**Left hemidiaphragm**			
Inspiratory excursion (QB)/Vti (mm/L/kg)	2.7 ± 0.9 [1.5–4.2]	2.6 ± 0.9 [1.6–4.2]	NS
Inspiratory velocity (QB)/Vti (mm/s/L/kg)	2.7 ± 1.3 [1.3–5.8]	2.4 ± 0.9 [1.3–4]	NS
Inspiratory excursion (DI)/Vimax (mm/ml/kg)	1.69 ± 0.5 [1.1–2.8]	1.68 ± 0.4 [1–2.3]	NS
Inspiratory thickening (QB)/Vti (%/L/kg)	4.4 ± 2.1 [2.2–8.4]	3.8 ± 2 [1.5–8.2]	<0.03
Thickening fraction (DI)/Vimax (%/L/kg)	3.9 ± 1.3 [2.1–6]	3.1 ± 1.2 [1.3–5.2]	<0.001

In patients with hemidiaphragm paralysis, on the paralyzed side, the excursion at the begining of inspiration was mostly nil or paradoxical therefore, the indices, using the excursion were close to zero. During inspiration, a lack of significant thickening (<20%) or thinning was observed and indices using the percentage thickening were also close to zero.

The results of the comparison of ultrasound parameters between subjects with NLF and the healthy side of patients with hemidiaphragm paralysis are reported in Tables 5, 6.

**TABLE 5:** Comparison of ultrasound markers of left hemidiaphragm between subjects with normal lung function (NLF) and patients with right hemidiaphragm paralysis (RHP).

	Subjects (NLF)	Patients (RHP)	*p*-Value
Men, women (% of women)	71 - 51 (42%)	17 – 14 (45%)	NS
Inspiratory excursion (QB) (cm)	2 ± 0.5	3.6 ± 0.9	<0.001
Inspiratory velocity (QB) (cm/s)	1.9 ± 0.63	3.7 ± 1.2	<0.001
Inspiratory excursion (DI) (cm)	5.5 ± 1	5.9 ± 1.2	NS
Inspiratory thickening (QB) %	31 ± 15	66 ± 26	<0.001
Thickening fraction (DI) %	110 ± 34	145 ± 40	<0.001
Vti (L)	0.58 ± 0.18	0.57 ± 0.2	NS
Vti (ml/kg)	8 ± 2	8 ± 3	NS
Vimax (L)	2.5 ± 0.8	1.5 ± 0.6	<0.001
Inspiratory excursion (QB)/Vti (mm/L)	37 ± 13	72 ± 32	<0.001
In mm/L/kg	2.7 ± 0.9	5 ± 2	<0.001
Inspiratory velocity (QB)/Vti (mm/s)	36 ± 16	78 ± 42	<0.001
In mm/s/kg	2.5 ± 1.1	5.3 ± 2.6	<0.001
Inspiratory excursion (DI)/ViMax (mm/L)	24 ± 6	43 ± 14	<0.001
In mm/L/kg	1.68 ± 0.45	3 ± 1	<0.001
Inspiratory thickening (QB)/VTi (%/L)	58 ± 33	139 ± 93	<0.001
In %/L/kg	4.1 ± 2.1	9.2 ± 5.1	<0.001
Thickening fraction/ViMax (%/L)	49 ± 21	112 ± 56	<0.001
In %/L/kg	3.4 ± 1.3	7.4 ± 2.8	<0.001

Vti, inspired volume during quiet breathing at tidal volume; Vimax, inspired volume during deep inspiration; QB, quiet breathing; DI, deep inspiration.

**TABLE 6:** Comparison of ultrasound markers of right hemidiaphragm between subjects with normal lung function (NLF) and patients with left hemidiaphragm paralysis (LHP).

	Subjects (NLF)	Patients (LHP)	*p*-Value
Men, women (percentage of women)	71–51 (42%)	24–13 (36%)	NS
Inspiratory excursion (QB) (cm)	2 ± 0.5	3 ± 0.6	<0.001
Inspiratory velocity (QB) (cm/s)	1.86 ± 0.7	2.8 ± 1	<0.001
Inspiratory excursion (DI) (cm)	5.4 ± 1	6 ± 1.3	<0.005
Inspiratory thickening (QB) %)	34 ± 17	60 ± 33	<0.001
Thickening fraction (DI) %	111 ± 44	134 ± 41	<0.005
Vti (L)	0.58 ± 0.19	0.54 ± 0.18	NS
Vti (ml/kg)	8.2 ± 2.5	7.6 ± 2.6	NS
Vimax (L)	2.5 ± 0.8	1.9 ± 0.6	<0.001
Inspiratory excursion (QB)/Vti (mm/L)	36 ± 11	62 ± 22	<0.001
In mm/L/kg	2.6 ± 0.8	4.5 ± 2	<0.001
Inspiratory velocity (QB)/Vti (mm/s)	34 ± 15	60 ± 36	<0.001
In mm/s/kg	2.47 ± 1.1	4.4 ± 3.2	<0.001
Inspiratory excursion (DI)/ViMax (mm/L)	23 ± 6	34 ± 10	<0.001
In mm/L/kg	1.63 ± 0.45	2.5 ± 0.8	<0.001
Inspiratory thickening (QB)/Vti (%/L)	63 ± 33	119 ± 70	<0.001
In %/L/kg	4.4 ± 2	8.6 ± 5.4	<0.001
Thickening fraction/ViMax (%/L)	49 ± 25	81 ± 43	<0.001
In %/L/kg	3.4 ± 1.6	5.7 ± 3	<0.001

Vti, inspired volume during quiet breathing at tidal volume; Vimax, inspired volume during deep inspiration; QB, quiet breathing; DI, deep inspiration.

### Results of the logistic regression analysis

The independent variables studied in the analysis were Inspiratory excursion (QB)/Vti, Inspiratory excursion (DI)/ViMax, Inspiratory thickening (QB)/Vti and Thickening fraction (DI)/ViMax.

Inspiratory Velocity (QB)/Vti was identified as a collinear variable and was not included in the analysis. The more pertinent ultrasound indice identified by the logistic regression analysis for the prediction of increased diaphragm activity was the ratio Inspiratory excursion (QB)/Vti (Tables 7, 8).

**TABLE 7:** Results of the logistic regression analysis in men.

Parameters	Estimate	Standard deviation	*p*-Value
Constant	16.13	2.9	<0.00001
Inspiratory excursion (QB)/Vti	−0.12	0.035	<0.001
Inspiratory excursion (DI)/ViMax	−0.24	0.079	0.002
Inspiratory thickening (QB)/Vti	−0.026	0.012	0.026
Thickening fraction (DI)/ViMax	−0.03	0.02	0.1

**TABLE 8:** Results of the logistic regression analysis in women.

Parameters	Estimate	Standard deviation	*p*-Value
Constant	11.83	2.29	<0.00001
Inspiratory excursion (QB)/Vti	−0.075	0.025	<0.005
Inspiratory excursion (DI)/ViMax	−0.086	0.054	0.11
Inspiratory thickening (QB)/Vti	−0.01	0.008	0.15
Thickening fraction (DI)/ViMax	−0.032	0.015	0.03

Hemi, hemidiaphragm; Insp, inspiratory; QB, quiet breathing; Vti, inspired volume during quiet breathing at tidal volume; Vimax, inspired volume during a deep inspiration.

In women, the positive and negative predictive values of this index were 89 and 90%, respectively. In men, the positive and negative predictive values were 79 and 91%.

## Discussion

In this work, we studied ultrasound diaphragm motion-volume indices. Diaphragm motion was evaluated using the amplitude or velocity of the excursion during the inspiratory phase during quiet breathing or deep inspiration. The percentage of inspiratory thickening was also tested during quiet breathing or deep inspiration.

In the population of subjects with normal lung function studied, as previously demonstrated (3, 4, 33), excursion and diaphragm thickness (12, 34) were different in men and in women. These differences were also observed when these parameters were divided by the breathing volume of the corresponding cycle. Therefore, normal values and limits of normality should be used according to sexe.

Since breathing volume is related to body mass, we also investigated the normal values of the indices divided by inspired volume and body mass. When the breathing volume was normalized to body mass, no gender differences were found for the indices combining diaphragm excursion and gas volume. This finding suggests that body mass has a significant impact on diaphragm excursions and participates to the differences between men and women.

By contrast, the indices using the inspiratory thickening (during quiet breathing or deep inspiration) remained significantely different between men and women after indexing the inspired volume to the body mass. This result was not suprising since although the breathing volume is commonly higher in men than in women, the percentage of thickening (during quiet breathing or deep inspiration) is not different between the sexes (5, 8, 12, 34).

The study of 122 subjects with normal lung function determined normal values and limits of normality for these various parameters. The use of the lower and upper limits of normality should be useful in assessing the effectiveness of diaphragm motion. To support this interest, in the second part of the study, we studied patients with hemidiaphragm paralysis.

On the paralyzed side, the mean ratio of amplitude (or velocity) of excursion divided by inspired volume (in absolute value or body mass normalized) was zero. Indeed, at quiet breathing, the motion was the more frequently nil. In some cases, the excursion was very weak or paradoxical. The use of the percentage of thickening also leads to a low index since the recognized criteria for diagnosing the hemidiaphragm paralysis is a percentage of thicknening less than 20% (11). In our population, the thickening fraction was most often nil. It was negative when a thinning was found. Various pathophysiologies could be involved in the hemidiaphragm dysfunction such as damage to the phrenic nerve or impaired muscle function.

The lower limit of normality for indices combining diaphragm excursion or thickening to maximal inspired volume could be useful for diagnosing diaphragm dysfunction. Indeed, it has been shown that some respiratory diseases such as COPD or lung fibrosis lead to a limitation of the excursion during deep inspiration (16, 18, 23). In such circumstances, it may be difficult to distinguish the limitation of excursion secondary to a decrease in inspiratory capacity from an impairment in diaphragm function. When the limitation is only related to the decrease in inspiratory capacity, such as in respiratory diseases with ventilatory defect, the ratio of excursion to deep inspiration divided by the inspired volume should remain normal on both sides. On the other hand, in patients suffering from dysfunction of an hemidiaphragm, an assymetry between the two sides must be observed with a normal indice on the healthy side and a decrease in the indice on the side suffering from dysfunction.

The comparison of the two sides was supported by measurements in patients with normal lung function. Indeed, the breathing volume divided by the diaphragm excursion during deep inspiration was similar on both sides, leading to an average ratio around 1 (see Table 3). Therefore, a dysfunction may be suspected when this ratio is outside the limits of normality (0.7–1.3 in women, 0.7–1.2 in men). This indice is particularly important in patients who were not able to repeat the same breathing volume during the ultrasound scanning.

As previously shown (12), on the healthy side of patients with hemidiaphragm paralysis, an increase in diaphragm activity was observed during quiet breathing. Indeed, as compared to subjects with normal lung function an increase in excursion and percentage of thickening was recorded. These findings suggest a compensatory mechanism (24) aiming to maintain appropriate gas exchanges despite the decrease of inspiratory work secondary to the hemidiaphragm paralysis. Since breathing volumes did not increase during quiet breathing in patients with hemidiaphragm paralysis compared to subjects with normal lung function, the ratio amplitude or velocity of excursions (in absolute value or indexed by body mass) divided by the inspired volume, increased (cf. Tables 5, 6). The indices using the percentage of thickening were also significantely increased. Consequently, during quiet breathing an indice higher than the upper value of normality, is in favor of an increase in diaphragm activity. It would be interesting to carry out further studies in patients suffering from lung diseases to assess the possible relationship between these indices and the severity of the disease.

It has been reported the interest of the ratio of respiratory rate to diaphragmatic excursion during the spontaneous breathing trial in predicting weaning outcome (35). Consequently, the weaning process of mechanical ventilation could also be an interesting context for assessing the predictive value of our indices. In addition, the simultaneous recordings of signal from the beginnning of inspiration, inspired volume and diaphragm motion, using the device developed by our team (32), could facilitate the detection of patient-ventilator asynchronies during non invasive ventilation (36). Furthers studies on these topics would be interesting.

**FIGURE 6:**
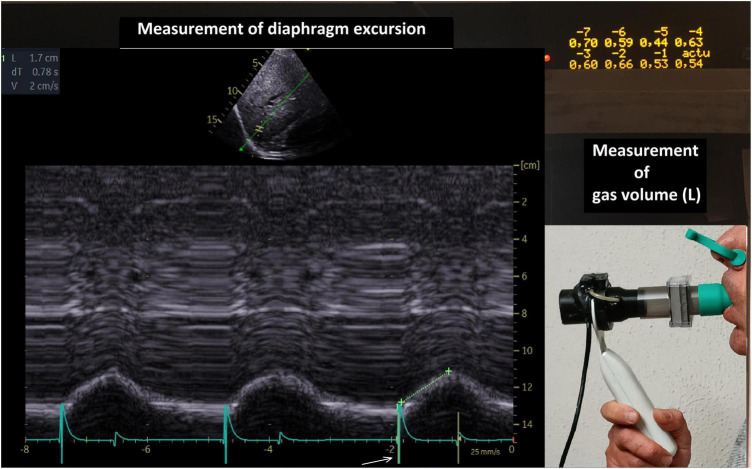
Simultaneous recording of diaphragm excursion and gas volume. The volumes of the last breathing cycles, from –7 to present (actu = 0.54 L) were recorded. The diaphragm excursion of the corresponding cycle was measured (here 1.7 cm during quiet breathing). Calculation of the indice inspired volume divided by diaphragm excursion during quiet breathing : 17/0.54 = 31.5 mm/L. Arrow = beginning of inspiration.

According to our statistical analysis the better indice allowing to detect diaphragm hyperactivity in both sexes was the ratio inspiratory excursion during quiet breathing divided by inspired volume (see Figure 6).

Some study limitations may be noted. In further studies, it would be important to increase the number of subjects with normal lung function included in order to improve the accuracy of normal values.

In our population of patients with hemidiaphragm dysfunction, it was not possible to be sure that the hemidiaphragm on the non paralyzed side was healthy. Nevertheless, the increase in diaphragm activity was statistically significant suggesting that the compensatory phenomenon was active and that most patients have good hemidiaphragmatic function on the non-paralyzed side.

It is important to note that while these indices should be informative in assessing the quality of the respiratory function, the study of the diaphragm function may not always reflect the work of breathing because in COPD patients (37) and in some circumstances such as during a spontaneous breathing trial (38) an overuse of the accessory muscles may be involved. Consequently, analysis of all inspiratory muscles should be important for accurate analysis of the work of breathing.

## Conclusion

Altough the normal values of diaphragmatic motion have been previously determined, a same motion, can generate different inspired volumes depending on the quality of the broncho-pulmonary system. The normal values of the ratio diaphragm motion divided by inspired volume, determined in our study, could be used to estimate the performance of the respiratory system integrating diaphragmatic function and thoraco-pulmonary system. In accordance with our hypothesis, the increase in the activity of the healthy side in patients with hemidiaphragm paralysis led to an increase in diaphragm motion-volume indices. Further studies would be interesting to assess the interest of these indices in patients with respiratory diseases.

## Data availability statement

The original contributions presented in this study are included in the article/supplementary material, further inquiries can be directed to the corresponding author.

## Ethics statement

The studies involving human participants were reviewed and approved by Ethics Board of the Marseilles Hospital Institution (registered under number 2RLK6P). The patients/participants provided their written informed consent to participate in this study.

## Author contributions

AB and FB conceived and designed the study. AM, MB, and SD assisted with the technical aspects of the protocol, recruited all the participants, and involved in the acquisition of the data. AB performed the ultrasound examinations. GC analyzed the data and performed the statistical analysis. AB, GC, and FB drafted the manuscript. AM and SD critically revised the manuscript for important intellectual content. All authors provided final approval of the version to be published.
